# Behavioral symptoms of dementia and psychotropic use during the COVID-19 pandemic

**DOI:** 10.3389/fepid.2026.1746012

**Published:** 2026-04-13

**Authors:** Jung Min Yoon, Kwame Kissi-Twum, Alison M. Trinkoff, T. Joseph Mattingly

**Affiliations:** 1 Division of Nursing, Ewha Womans University, Seoul, Republic of Korea; 2 Department of Pharmacotherapy, University of Utah College of Pharmacy, Salt Lake City, UT, United States; 3 School of Nursing, University of Maryland, Baltimore, MD, United States

**Keywords:** behavioral and psychological symptoms of dementia (BPSD), COVID-19, dementia, depression, psychotropic use

## Abstract

**Background:**

As nursing home settings and their residents were severely affected by the COVID-19 pandemic, changes in care practices along with increased social isolation during the pandemic may have increased the prevalence of behavioral and psychological symptoms of dementia (BPSD) and psychotropic medication use in nursing homes.

**Methods:**

We conducted a repeated cross-sectional study using 2019 and 2020 Minimum Data Set from nursing homes in New York, Utah, and Colorado. The primary outcome was a composite BPSD measure, defined as two or more symptoms among depression, wandering, rejection of care, hallucinations, delusions, or verbal/physical behavioral symptoms. Secondary outcomes included neuropsychiatric sub-syndromes and psychotropic use.

**Results:**

From 2019 to 2020, the prevalence of two or more BPSDs increased from 19.0% (95% CI, 18.8–19.2) to 20.2% (95% CI, 20.0–20.5). Increases in BPSD were largely driven by depressive symptoms, which increased by 63%, from 17.4% (95%CI, 17.1–17.6) in 2019 to 28.3% (95%CI, 28.0–28.5) in 2020. Whereas, the prevalence of psychosis-related symptoms changed minimally, from 5.6% (95%CI, 5.5–5.8) to 5.7% (95%CI, 5.5–5.8), and agitation-related symptoms decreased from 30.0% (95%CI, 29.8–30.3) in 2019 to 29.6% (95%CI, 29.4–30.0) in 2020. Antipsychotic and sedative use decreased while antidepressant and antianxiety use remained steady.

**Discussion:**

In this study of nursing home residents, the prevalence of BPSD was higher during the COVID-19 pandemic, primarily driven by increased depressive symptoms. These findings underscore the need to strengthen depression screening and mental health support for nursing home residents during public health emergencies such as pandemics.

## 1 Introduction

Behavioral and psychological symptoms of dementia (BPSD) refer to a group of symptoms related to cognitive impairment, including affective symptoms, psychosis, hyperactivity, euphoria, aberrant motor behaviors, disinhibition, eating disturbances, and sleep disorders. BPSD occur in nearly all individuals with dementia to some extent throughout their illness (1–3). BPSD are associated with worsening cognitive impairment and physical dysfunction in patients with dementia, (4) and impose additional physical and emotional burdens on caregivers.

BPSD are commonly evident in cognitively impaired residents residing in nursing homes (NHs), and prevalence estimates are often high when BPSD is defined as the presence of any symptom using comprehensive inventories and/or longer reference windows (e.g., cumulative occurrence over the course of illness) (5–7). Additionally, the reported prevalence of depressive symptoms varies widely across long-term care settings, depending on the population and measurement approach (8–11). First-line treatment for BPSD should consist of individualized non-pharmacologic interventions to manage behavioral symptoms before pharmacological interventions are used (2, 12). Successful implementation of these approaches requires adequate nurse staffing and high-quality care, as these frontline healthcare providers are crucial for assessing BPSD and providing person-centered care (13, 14). Multidisciplinary collaborative approaches between nurses, physicians, allied health clinicians, and behavioral therapists are also required to develop and implement optimal non-pharmacological interventions (15). Close contact and interactions between patients and their caregivers are major components of providing non-pharmacological interventions (1, 15).

The COVID-19 pandemic disproportionately affected older adults in NHs, with cases, hospitalizations, and deaths highly concentrated among older adults with underlying conditions. In the U.S., New York (NY) state was an initial epicenter of the pandemic, with the highest incidence, hospitalization rates, and mortality in New York City during the first 3 months (16). Because of the high proportion of older adults with moderate to severe cognitive impairment and multiple chronic illnesses, the pandemic greatly affected NH care quality (17–19). Physical/social distancing rules restricted the implementation of non-pharmacological interventions, and some were adapted to reduce infection risk (20). Restrictive measures needed for infection control increased social isolation, leading to worsened behavioral symptoms (21). Also, many NHs encountered challenges in providing high-quality dementia care due to nursing staff shortages, bans on family visits, limited telehealth services, and decreased support from behavioral care teams and special care professionals (22). Staff shortages limited the provision of appropriate clinical care and oversight, including medication management (20, 23).

Such changes in care practices from the pandemic may have led to an increased reliance on psychotropic use to control BPSD. However, studies assessing the prevalence of BPSD and psychotropic use among NH residents in the U.S. during the pandemic remain limited, particularly with regard to symptom-specific patterns. The purpose of this study was to compare the prevalence of BPSD and psychotropic medication use before and after the onset of the pandemic, with the hypothesis that the overall BPSD occurrence and psychotropic medication use would be higher during the pandemic period (the year of 2020) compared to the pre-pandemic period (the year of 2019).

## 2 Methods

### 2.1 Study design and participants

We conducted a repeated cross-sectional study using 2019 and 2020 Minimum Data Set (MDS) 3.0 data for nursing homes (NHs) and swing-bed providers (non-NHs with skilled nursing beds) in three states: New York (NY), Utah (UT), and Colorado (CO). MDS is a federally mandated assessment system that captures standardized clinical and functional information on all residents of Medicare- or Medicaid-certified long-term care facilities in the United States (24). MDS assessments provide detailed resident-level data on health status, cognitive and physical function, and psychosocial well-being. We included all residents with dementia from January 1, 2019 to December 31, 2020. Dementia status was identified using MDS 3.0 Section I diagnoses (Alzheimer's disease[I4200] or other dementia[I4800]). The institutional review board of the University of Utah determined that the study did not meet the criteria for human subjects research since there was no identifiable private information in the data or interactions with any individuals.

### 2.2 Study outcomes/variables

The primary outcome variable was a BPSD score, which was a composite of any occurrence of moderate to severe mood symptoms, wandering, rejection of care, hallucinations, delusions, verbal and physical behavioral symptoms. For each resident assessment, the MDS Section E captures the presence of behavioral symptoms, including wandering, rejection of care, hallucinations, delusions, and verbal and physical behaviors in the 7 days preceding the assessment. This was categorized into a binary variable defined as present if a resident experienced at least two of these symptoms.

In addition, the MDS Section D assessed mood symptoms using the Patient Healthcare Questionnaire (PHQ-9©). For this assessment, residents were asked if they had experienced a range of mood symptoms over the last two weeks before the assessment. For residents unable to complete the self-reported PHQ-9, the staff-observed version (PHQ-9 OV) was used, in which staff members rate observable mood symptoms over the same reference period (25, 26). PHQ-9 scores range from 0 to 27, with higher scores indicating greater severity; scores of 0–4 represent none or minimal symptoms, 5–9 mild, 10–14 moderate, 15–19 moderately severe, and 20–27 severe (27, 28). We derived a binary indicator from the total PHQ-9 score; scores ≥10 indicated at least moderate depressive symptoms.

We also created outcome variables to indicate the presence of neuropsychiatric subsyndrome for psychosis, agitation, and depression. These were derived by grouping BPSD symptoms that consistently co-occur during the course of dementia (7). Residents with hallucinations or delusions were classified under psychosis. Agitation included those with any reported instances of rejection of care, wandering, or physical or verbal abuse, and depression was defined as the presence of moderate to severe mood symptoms.

To evaluate the use of psychotropic medications, we recorded whether a resident received antidepressants, hypnotics, antipsychotics, and antianxiety medications in the look-back period.

### 2.3 Covariates

Covariates measured included cognitive impairment severity, physical function, pain, demographics (age, sex, race), medical comorbidities, and the number of assessments per resident. Cognitive impairment severity was measured using the Brief Interview for Mental Status scores (BIMS), with scores ranging from 0 to 15. Scores of 0–7 indicated severe impairment, 8–12 mild to moderate, and 13–15 represented intact cognition (29–31). For residents who did not complete the BIMS due to cognitive or communication limitations, the Cognitive Performance Scale (CPS) was used to measure cognitive impairment. The CPS ranges from 0 to 6, with 0–1 representing intact or borderline intact cognition, 2–3 indicating moderate impairment, and 4–6 indicating severe impairment (32, 33).

Physical function levels were assessed using the MDS 3.0 activities of daily living (ADL) items, which include bed mobility, walking, transfers, locomotion on unit, locomotion off unit, dressing, eating, toileting, personal hygiene, and bathing. For this analysis, physical impairment was quantified as the average number of ADL requiring at least limited assistance across all assessments (25, 34). To measure pain, residents were asked if they had experienced pain limiting daily activity over the past 5 days. Age, sex, and race were obtained from the MDS. Race was categorized as American Indian or Alaska Native, Asian, Black or African American, Native Hawaiian or Pacific Islander, White, and other. For each resident, we calculated the total number of comorbidities, which was used as a covariate (comorbidities included in the analyses are listed in Supplementary Table S1 in the supplemental online content). We included separate indicator variables for residents with schizophrenia, Tourette's syndrome, and Huntington's disease to adjust for their distinct neuropsychiatric profiles, which are associated with elevated behavioral symptom burden and differential psychotropic use patterns in long-term care settings. Finally, we counted the number of assessments per resident and included it as a covariate to account for differences in observation frequency and potential observation bias.

### 2.4 Statistical analysis

Sample characteristics and BPSD prevalence were described using frequencies and percentages. To assess the magnitude and direction of unadjusted differences in psychotropic use and BPSD between 2019 and 2020, we calculated standardized mean differences (SMDs). We used generalized estimating equations to compare the prevalence of BPSD in 2019 and 2020, i.e., before and during COVID-19. A logit link function with binary distributed errors was used to compare BPSD prevalence, after which we used predictive margins to estimate the adjusted BPSD prevalence by year. The model accounted for within-individual correlation across years and was adjusted for age, sex, race, medical comorbidities, the severity of cognitive impairment, physical function levels, pain, and the number of assessments.

In addition, subgroup analyses were performed by BPSD subsyndromes (psychosis, agitation, depression). We also assessed the correlations among the individual symptoms comprising the composite BPSD score using tetrachoric correlation coefficients to identify symptom clusters.

As part of the sensitivity analysis, we evaluated whether patterns of mild to severe mood symptoms (PHQ-9 > 4) and the prevalence of having at least one BPSD (≥1 symptom) differed between 2019 and 2020. This approach aligns more closely with studies that define BPSD as the presence of any symptom. Analyses were conducted using Stata version 18.0 (StataCorp) and R statistical software (v 4.4.3).

## 3 Results

Our analyses included 113,276 residents in 2019 and 104,563 in 2020. Table 1 shows the characteristics of NH residents with dementia in each year. The median age of residents was 85 years (IQR, 13) in 2019 and 84 years (IQR, 13) in 2020. Severe cognitive impairment was higher in 2020 (57.9%) than in 2019 (56.7%).

**Table 1:** Comparison of nursing home resident characteristics in 2019 and 2020.

Variable	2019	2020	Absolute standardized mean difference
Sample size	*N* = 113,276	*N* = 104,563	
Age, median (IQR)	85 (13)	84 (13)	0.025
Sex			0.002
Female	72,768 (64.2)	67,072 (64.1)	
Male	40,508 (35.8)	37,491 (35.9)	
Race			0.024
American Indian/Alaska Native	285 (0.3)	263 (0.3)	
Asian	3,369 (3.0)	3,053 (2.9)	
Black	16,312 (14.4)	15,585 (14.9)	
Native Hawaiian or Pacific Islander	154 (0.1)	132 (0.1)	
White	78,905 (69.7)	71,809 (68.7)	
Other	14,251 (12.6)	13,721 (13.1)	
States			0.005
New York	94,409 (83.3)	87,085 (83.3)	
Utah	4,244 (3.7)	3,838 (3.7)	
Colorado	14,623 (12.9)	13,640 (13.0)	
Cognition			0.040
Intact cognition	19,352 (17.1)	16,341 (15.6)	
Moderate cognitive impairment	29,703 (26.2)	27,700 (26.5)	
Severe cognitive impairment	64,221 (56.7)	60,522 (57.9)	
Presence of pain (% any pain in past 5 days)	33,127 (29.2)	28,142 (26.9)	0.052
Number of comorbidities, median (IQR)	6 (4)	6 (5)	0.166
Number of assessments per resident, median (IQR)	4 (3)	3 (4)	0.163
Physical function (mean number of ADL)	7.89 (2.22)	7.53 (2.26)	0.162

Data are presented as number (percentage). Means are presented with standard deviations (SD) in parentheses, and medians with interquartile ranges (IQR) in parentheses. An ASMD (absolute standardized mean difference) less than 0.1 is an indicator of negligible difference between years. Source: authors' analyses of data from MDS v3.0, 2019–2020. IQR, interquartile range; ADL, activities of daily living.

Table 2 presents the unadjusted prevalence of BPSD and psychotropic use in each year. The largest increase was observed in moderate to severe mood symptoms, which had an unadjusted prevalence of 16.9% in 2019 and 28.3% in 2020.

**Table 2:** Bivariate statistics comparing 2019 and 2020 unadjusted prevalence of psychotropic use and behavioral symptoms of dementia (BPSD) in nursing home residents.

Variable	2019 *N* (%)	2020 *N* (%)	Standardized mean difference
	*N* = 113,276 (%)	*N* = 104,563 (%)	
Psychotropic medications taken, past 7 days			
Antipsychotic medications	26,717 (23.6)	24,736 (23.7)	0.002
Antidepressants	56,727 (50.1)	52,132 (49.9)	−0.004
Hypnotics/sedatives	1,556 (1.4)	1,197 (1.1)	−0.021
Antianxiety medications	16,973 (15.0)	15,362 (14.7)	−0.008
BPSD (*n*, % experiencing these)			
Wandering	11,329 (10.0)	10,878 (10.4)	0.013
Verbal abuse	16,402 (14.5)	14,311 (13.7)	−0.023
Physical abuse	12,888 (11.4)	10,777 (10.3)	−0.034
Resists Care	20,419 (18.0)	17,954 (17.2)	−0.022
Hallucinations	2,672 (2.4)	2,703 (2.6)	0.015
Delusions	5,318 (4.7)	4,868 (4.7)	0.002
Mood symptoms (moderate to severe)	18,974 (16.9)	29,459 (28.3)	0.277

Frequencies represent the prevalence of psychotropic medication use and BPSD in 2019 and 2020. Source: authors' analyses of data from MDS v3.0, 2019–2020. BPSD, behavioral and psychological symptoms of dementia.

**Figure 1:**
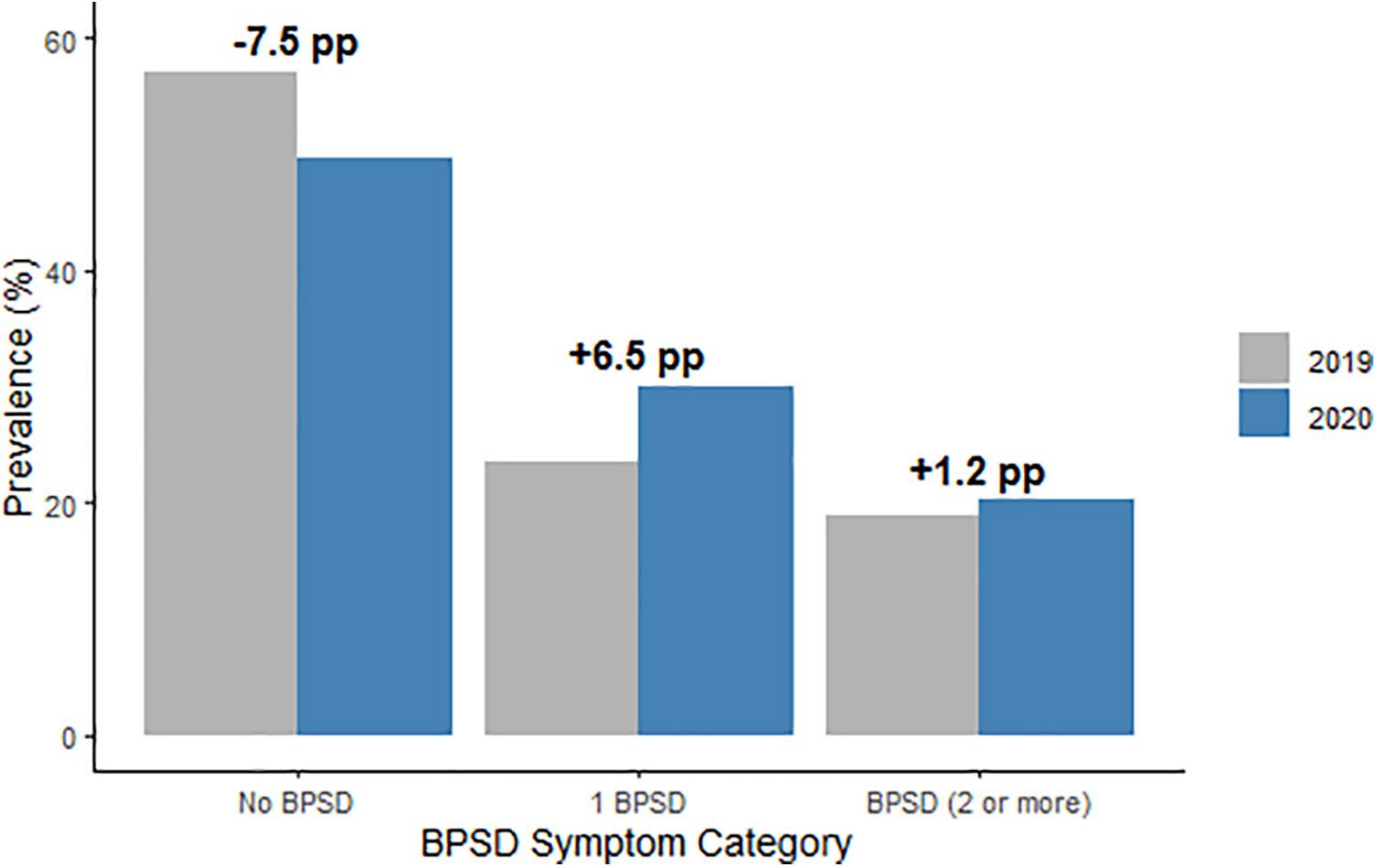
Prevalence of behavioral and psychological symptoms of dementia (BPSD) among nursing home residents with dementia before (2019) and after (2020) the onset of the COVID-19 pandemic. Bars represent the percentage of residents in each BPSD symptom category: no symptoms, exactly one symptom, or two or more symptoms.

From 2019 to 2020, there was a 6.3% increase in the prevalence of having 2 or more BPSD, with a prevalence of 2 or more BPSD in 2019 of 19.0% (95%CI, 18.8–19.2) and 20.2% (95%CI, 20.0–20.5) in 2020 (Table 3). A decrease in the proportion of residents who had no BPSD was also observed (Figure 1). In addition, antipsychotic use decreased by 1.3 percentage points, hypnotic/sedative use declined slightly, and antidepressant and antianxiety medication use remained stable (Table 4).

**Table 3:** Prevalence of BPSD in 2019 vs. 2020 (estimated effect of the COVID-19 pandemic).

Variable	2019 (%)	2020 (%)	Prevalence difference (95% CI)	Percentage increase
Composite				
BPSD ≥ 2	19.0 (18.8–19.2)	20.2 (20.0–20.5)	1.2 (0.9–1.5)	6.3
1 BPSD	23.5 (23.2–23.7)	30.0 (29.7–30.3)	6.5 (6.2–6.9)	27.7
No BPSD	57.2 (56.9–57.5)	49.7 (49.4–50.0)	−7.5 (−7.9 to −7.2)	-
BPSD subsyndromes				
Mood Alone	17.4 (17.1–17.6)	28.3 (28.0–28.5)	10.9 (10.6–11.2)	62.6
Psychosis	5.6 (5.5–5.8)	5.7 (5.5–5.8)	0.03 (−0.1 to 0.2)	0.89
Agitation	30.0 (29.8–30.3)	29.6 (29.4–30.0)	−0.4 (−0.6 to −0.03)	-

Prevalence differences represent percentage point changes and were estimated using marginal predictions from generalized estimating equations (GEE), accounting for within-resident correlation across years, and adjusting for age, sex, race, comorbidities, cognitive function, physical function, number of assessments, and pain. Psychosis indicates the presence of delusions or hallucinations. Agitation includes rejection of care, wandering, physical, and verbal abuse. “Mood alone” refers to the presence of moderate to severe symptoms of depression. Source: authors' analyses of data from MDS v3.0, 2019–2020. BPSD, behavioral and psychological symptoms of dementia.

**Table 4:** Prevalence of psychotropic use among nursing home residents with dementia in 2019 vs. 2020.

Variable	2019 (%)	2020 (%)	Prevalence difference (95% CI)
Hypnotics/sedatives	1.3 (1.2–1.4)	1.1 (1.0–1.2)	**−0.21** **(−0.3 to −0.1)**
Antianxiety medications	15.0 (14.8–15.2)	14.9 (14.7–15.1)	−0.07 (−0.2 to 0.1)
Antipsychotic medications	24.6 (24.4–24.9)	23.3 (23.1–23.5)	**−1.3** **(−1.6 to −1.1)**
Antidepressants	49.6 (49.3–49.8)	49.8 (49.5–50.1)	0.2 (−0.1 to 0.4)

Prevalence differences represent percentage point changes and were estimated using marginal predictions from generalized estimating equations (GEE), accounting for within-resident correlation across years, and adjusting for age, sex, race, comorbidities, cognitive function, physical function, number of assessments, and pain.

Bold values indicate statistically significant prevalence differences at the 95% confidence level.

**Figure 2:**
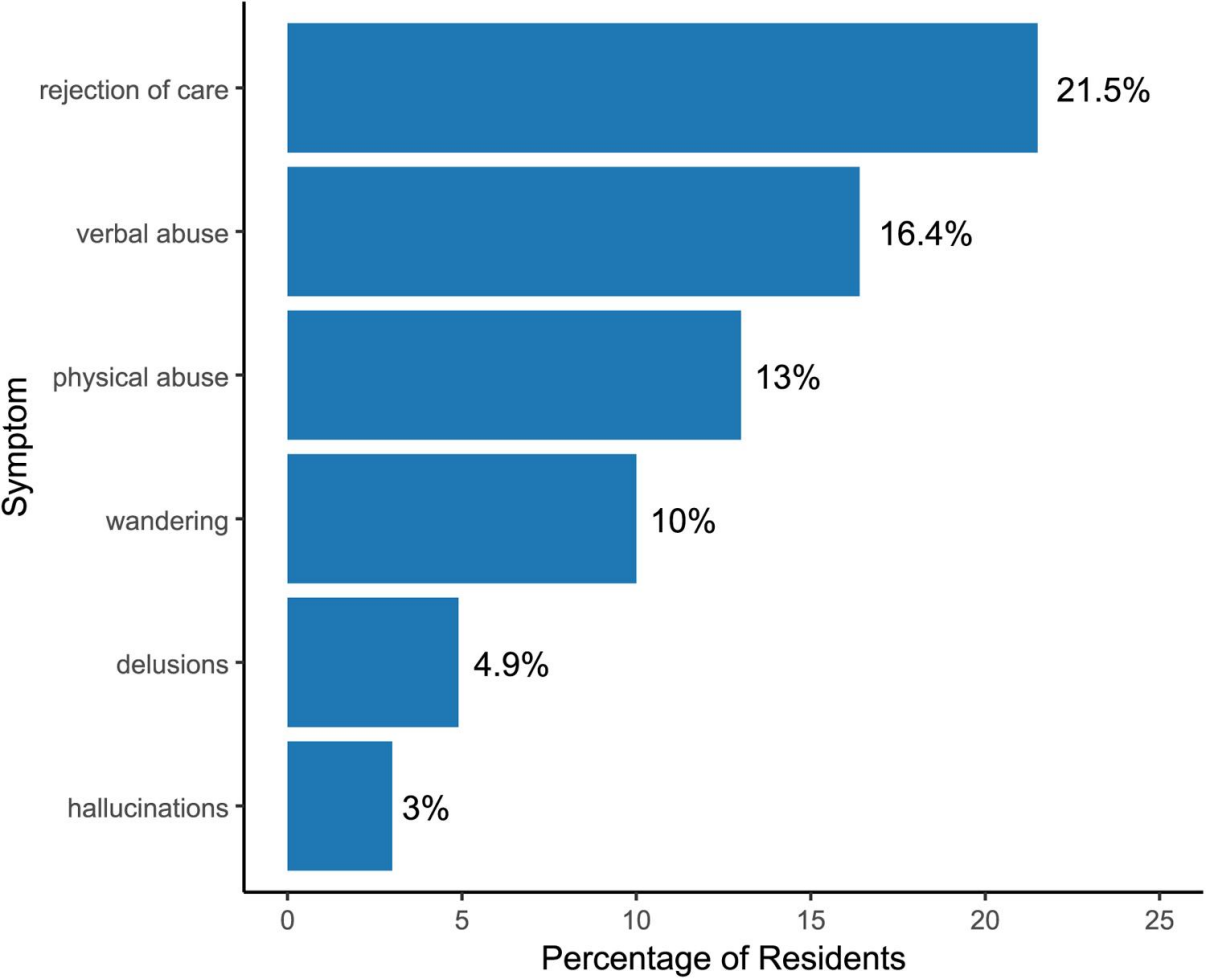
Symptom co-occurrence among residents with moderate to severe depressive symptoms. The most frequent co-occurring symptom among those with two or more BPSD was a combination of depressive symptoms and resisting care, followed by combinations of depression and verbal abusive symptoms, and depression and physical abusive symptoms.

In analyses grouped by BPSD subsyndrome, the largest increase was observed in mood-related symptoms (depression). The prevalence of moderate to severe depression increased by 63%, from 17.4% (95% CI, 17.1–17.6) in 2019 to 28.3% (95% CI, 28.0–28.5) in 2020. Whereas, the prevalence of psychosis-related symptoms changed minimally, from 5.6% (95% CI, 5.5–5.8) to 5.7% (95% CI, 5.5–5.8), and agitation-related symptoms decreased from 30.0% (95% CI, 29.8–30.3) in 2019 to 29.6% (95% CI, 29.4–30.0) in 2020. As depicted in Figure 2, among residents with moderate to severe mood symptoms, the most common co-occurring symptoms were rejection of care (21.5%), verbal abuse (16.4%), and physical abuse (13%).

In sensitivity analysis, there was a 29% increase in the prevalence of mild-to-severe mood symptoms, from 39.5% (95% CI, 39.3–39.8) in 2019 to 51.1% (95% CI, 50.8–51.4) in 2020. In addition, the prevalence of having at least one BPSD was 42.7% (95% CI, 42.5–43.0) in 2019 and 50.3% (95% CI, 50.0–50.6) in 2020.

**Figure 3:**
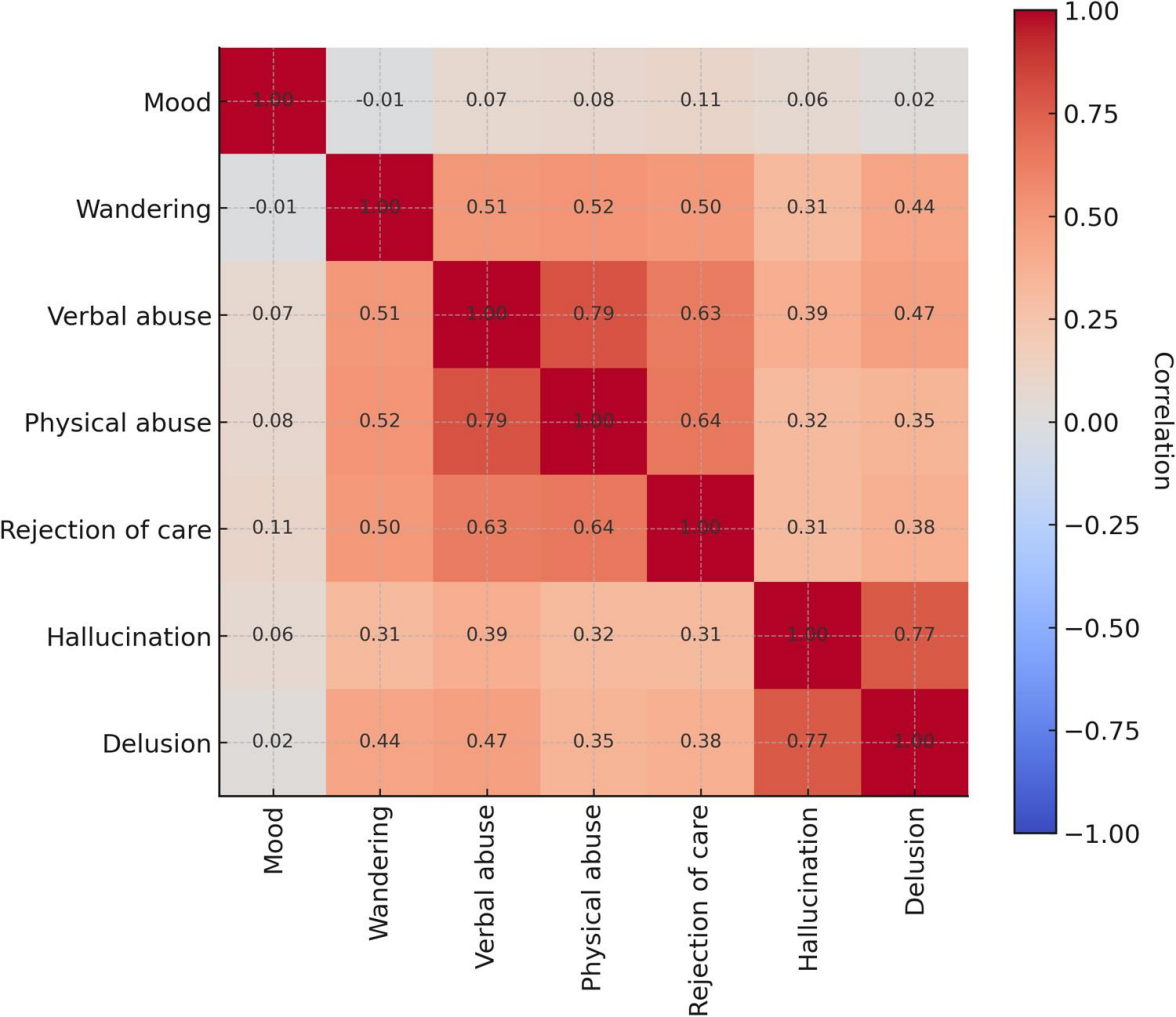
Tetrachoric correlation matrix. In the analysis, each BPSD symptom assessed in the MDS was treated as a binary indicator reflecting whether a resident exhibited a clinically significant behavior during a defined look-back period. These binary indicators are assumed to reflect underlying continuous latent traits such as mood disturbance, psychotic symptom severity, or behavioral dysregulation, which are discretized based on clinical observation thresholds. Under this conceptualization, the use of tetrachoric correlations is appropriate, as it estimates the correlation between the underlying continuous variables that give rise to the observed binary responses.

Tetrachoric correlations indicated that certain BPSD symptoms clustered together (Figure 3). Verbal and physical abuse were highly correlated (r = 0.79), and both were moderately associated with rejection of care (r = 0.63–0.64). Hallucinations and delusions were also highly correlated (r = 0.77). In contrast, mood symptoms showed weak correlations with other BPSD domains (r values ≤0.11).

## 4 Discussion

Our study indicated a modest increase in the prevalence of BPSD manifestation among older adults with dementia between 2019 and 2020. During 2020, a slight increase in the percentages of older adults with one or two or more BPSD symptoms was found. Importantly, findings were robust to the outcome definition: when we applied alternative thresholds (≥1 BPSD symptom and PHQ-9 > 4). Prevalence estimates increased as expected, although changes across 2019–2020 remained modest in the same direction. Restriction measures during the pandemic may have been related to changes in BPSD manifestation, notably the increase was most pronounced for mood symptoms.

Pandemic-related changes were not necessarily expected to increase all BPSD domains in parallel. Regarding BPSD subsyndromes, compared to depressive symptoms, which had a significant increase during the pandemic, the prevalence of psychosis remained stable (or was only slightly higher), and the agitation symptom groups showed a small decrease from 2019 to 2020. Reduced visitation and fewer structured activities may have worsened mood-related symptoms through greater social isolation and reduced psychosocial supports (35–37). Disruptions to routines, changes in care staff, and PPE-related communication barriers may have increased distress or psychotic symptoms for some individuals (36), whereas reduced group activities/visitors and lower levels of untargeted stimulation may reduce certain triggers for psychotic or agitated behaviors in some residents (38).

In addition, the effect of the restriction may have varied by cognitive function levels. In this study, most residents had severe cognitive impairment, and their psychosis and agitation may have been less influenced by lockdown compared to those with mild impairment. Previous studies reported a lower prevalence of psychosis and agitation/aggression among those with more advanced dementia compared to those with mild dementia during the pandemic (38, 39). A lack of stimuli could rather benefit those with severe dementia in reducing the occurrence of psychosis and agitation (38, 40).

The most frequent co-occurring symptom among those with two or more BPSD was a combination of depressive symptoms and resisting care, followed by combinations of depression and verbal abusive symptoms, and depression and physical abusive symptoms. Other studies have reported a greater occurrence of resistance to care related to the presence of depressive symptoms among NH residents with dementia (41, 42). During the pandemic, lack of social interaction and limited provision of consistent quality care due to dramatic changes in activities and routines, and staff shortages in NHs may have led to resistance to care (43, 44). Prior studies highlighted that depression and physical/verbal aggression often co-occurred, and cognitive impairment and depression were related to greater occurrence of aggression (42, 45).

Although it was slight, an increased occurrence of wandering was noted during the pandemic, and this could be related to environmental distress among those with cognitive impairment (39, 46). Increased wandering may be attributed to frequent nighttime wandering, as boredom from reduced social interactions and limited medical care provision may have increased daytime napping and disrupted circadian rhythms (46–50). A slight increase in hallucinations and delusions also occurred during the pandemic. The provision of non-pharmacological interventions, such as cognitive rehabilitation, physical activities, and sensory stimulation, was disturbed during the lockdown, which may have triggered imagined stimuli and hallucinations (51, 52).

The study showed that antidepressant drug use among dementia residents remained steady, while antipsychotic drug use decreased. Although previous studies reported increases in both antipsychotic and antidepressant use during the pandemic (53–56), our findings differ partly because our outcome measured annual prevalence among all residents with dementia (a mix of long- and short-stay), whereas prior work tracked monthly trajectories among long-stay residents and observed increases during the pandemic months. Also, the decrease in antipsychotic use may have been partly influenced by policies from the Center for Medicare and Medicaid Services (CMS). For example, the CMS National Partnership to Improve Dementia Care in Nursing Homes, launched in 2012, has been associated with a nationwide reduction of antipsychotic use across NH residents through the pre-pandemic period (57). Antipsychotics can be used for psychosis, agitation, and aggression that are no longer responsive to non-pharmacological interventions; however, their use should be minimized to prevent adverse events, including anticholinergic effects, extrapyramidal symptoms, and sedation (58–60). During the study period, antidepressants were the most frequently used psychotropic agents. Antidepressants are often preferred over antipsychotics in older adults with dementia because they are generally better tolerated and may provide some benefit for depression, agitation, and aggression (61, 62). However, the use of antidepressants should be prudent due to increased risk of falls, hospitalization, and death (63).

Whereas the study indicated a decrease in the use of hypnotics/sedatives and a non-significant decrease in antianxiety drug use, supported by prior studies, (56, 64) the use of antianxiety drugs, including benzodiazepines, may have been avoided where possible due to concerns about dependence and withdrawal reactions for long-term use (65, 66). Although benzodiazepines can be used for agitated behaviors and sleep disturbances in patients with dementia, their use should be limited to short-term, severe acute distress (66–68).

### 4.1 Limitation

The current study has some limitations. Our sample came from three states, which may limit the generalizability of our findings to other US regions. Because we examined associations between BPSD prevalence and psychotropic drug use and the pandemic using a repeated cross-sectional design, causal conclusions cannot be made. Underlying year-to-year variation and longer-term pre-pandemic trends may have contributed to changes in BPSD prevalence and psychotropic use. However, the immediate temporal alignment with the pandemic supports interpreting these findings as pandemic-era changes rather than gradual background variation. As we analyzed only two years of data, we could not examine additional pre-pandemic years (e.g., 2017–2018) to distinguish pandemic-period changes from background variations. We did not directly measure facility-level pandemic policies (e.g., timing/intensity of visitation restrictions, activity cancellations, PPE practices, or cohorting), staffing disruptions, or changes in access to non-pharmacological interventions and resident social stimulation; thus, changes in BPSD subsyndromes should be interpreted cautiously and viewed as plausible explanations rather than causal conclusions. Previous studies have documented substantial underreporting and inconsistency in federal data on confirmed COVID-19 infection and disease severity during early 2020 (69, 70). Attempting to model infection-specific effects under these conditions may introduce misclassification bias. Accordingly, we focused on broader pandemic-period exposure.

In addition, because mood assessment relied on patient recall over the preceding two weeks; responses may be subject to recall bias or be influenced by the resident's current mood state. Changes in the severity of behavioral symptoms during the pandemic could not be assessed because the MDS records frequency but does not provide symptom intensity. The data also lacked information about changes in psychotropic dosages, so the analysis could not account for variations in dose adjustments.

## 5 Conclusion

Overall, our findings suggest the need for routine depression screening and access to mental health resources to address moderate to severe depression in long-term care settings, particularly during periods when care routines and social engagement are disrupted. Changes in prescribing patterns point to stable psychotropic use despite increased depression, emphasizing the need for stronger nonpharmacologic and psychosocial supports in long-term care. Policies that promote non-pharmacological interventions to address social isolation and emotional distress could ensure the quality of dementia care during future pandemic crises.

## Data Availability

The data analyzed in this study is subject to the following licenses/restrictions: The dataset analyzed in this study was obtained from the Centers for Medicare & Medicaid Services (CMS) under a data use agreement and is not publicly available. Researchers may request access to the Minimum Data Set (MDS) 3.0 through the Research Data Assistance Center (ResDAC) at https://resdac.org. Requests to access these datasets should be directed to https://resdac.org.

## References

[1] Bessey LJ Walaszek A. Management of behavioral and psychological symptoms of dementia. Curr Psychiatry Rep. (2019) 21(8):66. 10.1007/s11920-019-1049-5 31264056

[2] Kales HC Gitlin LN Lyketsos CG. Assessment and management of behavioral and psychological symptoms of dementia. BMJ. (2015) 350:h369. 10.1136/bmj.h369 25731881 PMC4707529

[3] van der Linde RM Brayne C Dening T. Depression and other behavioral and psychological symptoms of dementia–separate research worlds in need of a common understanding. Int Psychogeriatr. (2014) 26(2):177–83. 10.1017/S1041610213001592 24152758

[4] van der Linde RM Dening T Stephan BC Prina AM Evans E Brayne C. Longitudinal course of behavioural and psychological symptoms of dementia: systematic review. Br J Psychiatry. (2016) 209(5):366–77. 10.1192/bjp.bp.114.148403 27491532 PMC5100633

[5] Song JA Oh Y. The association between the burden on formal caregivers and behavioral and psychological symptoms of dementia (BPSD) in Korean elderly in nursing homes. Arch Psychiatr Nurs. (2015) 29(5):346–54. 10.1016/j.apnu.2015.06.004 26397440

[6] Sefcik JS Madrigal C Heid AR Molony SL Van Haitsma K Best I Person-centered care plans for nursing home residents with behavioral and psychological symptoms of dementia. J Gerontol Nurs. (2020) 46(11):17–27. 10.3928/00989134-20201012-03 33095889 PMC8274316

[7] Cerejeira J Lagarto L Mukaetova-Ladinska EB. Behavioral and psychological symptoms of dementia. Front Neurol. (2012) 3:73. 10.3389/fneur.2012.00073 22586419 PMC3345875

[8] Gruber-Baldini AL Zimmerman S Boustani M Watson LC Williams CS Reed PS. Characteristics associated with depression in long-term care residents with dementia. Gerontologist. (2005) 45(1):50–5. 10.1093/geront/45.suppl_1.50 16230749

[9] Matos Queiros A von Gunten A Martins M Wellens NIH Verloo H. The forgotten psychopathology of depressed long-term care facility residents: a call for evidence-based practice. Dement Geriatr Cogn Dis Extra. (2021) 11(1):38–44. 10.1159/000514118 33790939 PMC7989823

[10] Iden KR Engedal K Hjorleifsson S Ruths S. Prevalence of depression among recently admitted long-term care patients in Norwegian nursing homes: associations with diagnostic workup and use of antidepressants. Dement Geriatr Cogn Disord. (2014) 37(3-4):154–62. 10.1159/000355427 24157730

[11] Morley JE. Depression in nursing home residents. J Am Med Dir Assoc. (2010) 11(5):301–3. 10.1016/j.jamda.2010.03.012 20511093

[12] Davies SJ Burhan AM Kim D Gerretsen P Graff-Guerrero A Woo VL Sequential drug treatment algorithm for agitation and aggression in Alzheimer's and mixed dementia. J Psychopharmacol. (2018) 32(5):509–23. 10.1177/0269881117744996 29338602 PMC5944080

[13] Feast AR White N Candy B Kupeli N Sampson EL. The effectiveness of interventions to improve the care and management of people with dementia in general hospitals: a systematic review. Int J Geriatr Psychiatry. (2020) 35(5):463–88. 10.1002/gps.5280 32011033

[14] Wolf D Rhein C Geschke K Fellgiebel A. Preventable hospitalizations among older patients with cognitive impairments and dementia. Int Psychogeriatr. (2019) 31(3):383–91. 10.1017/S1041610218000960 30221613

[15] Grand JH Caspar S Macdonald SW. Clinical features and multidisciplinary approaches to dementia care. J Multidiscip Healthc. (2011) 4:125–47. 21655340 10.2147/JMDH.S17773 PMC3104685

[16] Thompson CN Baumgartner J Pichardo C Toro B Li L Arciuolo R COVID-19 outbreak - New York City, February 29-June 1, 2020. MMWR Morb Mortal Wkly Rep. (2020) 69(46):1725–9. 10.15585/mmwr.mm6946a2 33211680 PMC7676643

[17] McMichael TM Clark S Pogosjans S Kay M Lewis J Baer A COVID-19 in a long-term care facility - King County, Washington, February 27-March 9, 2020. MMWR Morb Mortal Wkly Rep. (2020) 69(12):339–42. 10.15585/mmwr.mm6912e1 32214083 PMC7725515

[18] Iaboni A Cockburn A Marcil M Rodrigues K Marshall C Garcia MA Achieving safe, effective, and compassionate quarantine or isolation of older adults with dementia in nursing homes. Am J Geriatr Psychiatry. (2020) 28(8):835–8. 10.1016/j.jagp.2020.04.025 32430111 PMC7196899

[19] Lester PE Holahan T Siskind D Healy E. Policy recommendations regarding skilled nursing facility management of coronavirus 19 (COVID-19): lessons from New York State. J Am Med Dir Assoc. (2020) 21(7):888–92. 10.1016/j.jamda.2020.05.058 32674814 PMC7264021

[20] Keng A Brown EE Rostas A Rajji TK Pollock BG Mulsant BH Effectively caring for individuals with behavioral and psychological symptoms of dementia during the COVID-19 pandemic. Front Psychiatry. (2020) 11:573367. 10.3389/fpsyt.2020.573367 33132936 PMC7574608

[21] Santini ZI Jose PE York Cornwell E Koyanagi A Nielsen L Hinrichsen C Social disconnectedness, perceived isolation, and symptoms of depression and anxiety among older Americans (NSHAP): a longitudinal mediation analysis. Lancet Public Health. (2020) 5(1):e62–70. 10.1016/S2468-2667(19)30230-0 31910981

[22] Davidson PM Szanton SL. Nursing homes and COVID-19: we can and should do better. J Clin Nurs. (2020) 29(15-16):2758–9. 10.1111/jocn.15297 32281165 PMC7262177

[23] McGarry BE Grabowski DC Barnett ML. Severe staffing and personal protective equipment shortages faced by nursing homes during the COVID-19 pandemic. Health Aff (Millwood). (2020) 39(10):1812–21. 10.1377/hlthaff.2020.01269 32816600 PMC7598889

[24] Centers for Medicare & Medicaid Services. Long term care minimum data set (MDS) 3.0. Available online at: https://resdac.org/cms-data/files/mds-30 (Accessed April 01, 2025).

[25] Centers for Medicare & Medicaid Services. Long-term care facility resident assessment instrument 3.0 User's Manual (2017). Available online at: https://downloads.cms.gov/files/mds-30-rai-manual-v115-october-2017.pdf (Accessed June 05, 2025). 26110197

[26] Saliba D DiFilippo S Edelen MO Kroenke K Buchanan J Streim J. Testing the PHQ-9 interview and observational versions (PHQ-9 OV) for MDS 3.0. J Am Med Dir Assoc. (2012) 13(7):618–25. 10.1016/j.jamda.2012.06.003 22796361

[27] Kroenke K Spitzer RL Williams JB. The PHQ-9: validity of a brief depression severity measure. J Gen Intern Med. (2001) 16(9):606–13. 10.1046/j.1525-1497.2001.016009606.x 11556941 PMC1495268

[28] Levis B Benedetti A Thombs BD, Collaboration DESD. Accuracy of Patient Health Questionnaire-9 (PHQ-9) for screening to detect major depression: individual participant data meta-analysis. BMJ. (2019) 365:l1476. 10.1136/bmj.l1476 30967483 PMC6454318

[29] Centers for Medicare & Medicaid Services. Long-term care facility resident assessment instrument 3.0 User's Manual version 1.15 (2017). Available online at: https://downloads.cms.gov/files/mds-30-rai-manual-v115-october-2017.pdf (Accessed June 05, 2025). 26110197

[30] Chodosh J Edelen MO Buchanan JL Yosef JA Ouslander JG Berlowitz DR Nursing home assessment of cognitive impairment: development and testing of a brief instrument of mental status. J Am Geriatr Soc. (2008) 56(11):2069–75. 10.1111/j.1532-5415.2008.01944.x 19016941

[31] Saliba D Buchanan J Edelen MO Streim J Ouslander J Berlowitz D MDS 3.0: brief interview for mental status. J Am Med Dir Assoc. (2012) 13(7):611–7. 10.1016/j.jamda.2012.06.004 22796362

[32] Morris JN Fries BE Mehr DR Hawes C Phillips C Mor V MDS cognitive performance scale. J Gerontol. (1994) 49(4):M174–82. 10.1093/geronj/49.4.M174 8014392

[33] Thomas KS Dosa D Wysocki A Mor V. The minimum data set 3.0 cognitive function scale. Med Care. (2017) 55(9):e68–e72. 10.1097/MLR.0000000000000334 25763665 PMC4567556

[34] Carpenter GI Hastie CL Morris JN Fries BE Ankri J. Measuring change in activities of daily living in nursing home residents with moderate to severe cognitive impairment. BMC Geriatr. (2006) 6:7. 10.1186/1471-2318-6-7 16584565 PMC1522014

[35] El Haj M Altintas E Chapelet G Kapogiannis D Gallouj K. High depression and anxiety in people with Alzheimer's disease living in retirement homes during the COVID-19 crisis. Psychiatry Res. (2020) 291:113294. 10.1016/j.psychres.2020.113294 32763552 PMC7357507

[36] Mulla RT Hirdes JP Kroetsch B McAiney C Heckman GA. Consequences of loneliness/isolation and visitation restrictions on the mood of long-term care residents without severe dementia pre-COVID-19 and during COVID-19: a scoping review. BMJ Open. (2025) 15(3):e090522. 10.1136/bmjopen-2024-090522 40139711 PMC11950938

[37] Simard J Volicer L. Loneliness and isolation in long-term care and the COVID-19 pandemic. J Am Med Dir Assoc. (2020) 21(7):966–7. 10.1016/j.jamda.2020.05.006 32505516 PMC7205644

[38] Knippenberg IAH Leontjevas R Nijsten JMH Bakker C Koopmans R Gerritsen DL. Stimuli changes and challenging behavior in nursing homes during the COVID-19 pandemic. BMC Geriatr. (2022) 22(1):142. 10.1186/s12877-022-02824-y 35183123 PMC8857739

[39] Leontjevas R Knippenberg IAH Smalbrugge M Plouvier AOA Teunisse S Bakker C Challenging behavior of nursing home residents during COVID-19 measures in the Netherlands. Aging Ment Health. (2021) 25(7):1314–9. 10.1080/13607863.2020.1857695 33291991

[40] Garcia LJ Hebert M Kozak J Senecal I Slaughter SE Aminzadeh F Perceptions of family and staff on the role of the environment in long-term care homes for people with dementia. Int Psychogeriatr. (2012) 24(5):753–65. 10.1017/S1041610211002675 22265186

[41] Galindo-Garre F Volicer L van der Steen JT. Factors related to rejection of care and behaviors directed towards others: a longitudinal study in nursing home residents with dementia. Dement Geriatr Cogn Dis Extra. (2015) 5(1):123–34. 10.1159/000369158 25999979 PMC4439779

[42] Kang B Pan W Karel MJ McConnell ES. Rejection of care and aggression among older veterans with dementia: the influence of background factors and interpersonal triggers. J Am Med Dir Assoc. (2021) 22(7):1435–41 e1. 10.1016/j.jamda.2021.03.032 33939963

[43] Backhouse T Killett A Mioshi E Khondoker M. What are the factors associated with people with advanced dementia refusing assistance with personal care? Int J Geriatr Psychiatry. (2023) 38(1):e5857. 10.1002/gps.5857 36490270 PMC10107826

[44] Gjellestad A Oksholm T Alvsvag H Bruvik F. Trust-building interventions to home-dwelling persons with dementia who resist care. Nurs Ethics. (2023) 30(7-8):975–89. 10.1177/09697330211041745 35189756 PMC10710005

[45] Majic T Pluta JP Mell T Treusch Y Gutzmann H Rapp MA. Correlates of agitation and depression in nursing home residents with dementia. Int Psychogeriatr. (2012) 24(11):1779–89. 10.1017/S104161021200066X 22591584

[46] Kuroda Y Sugimoto T Matsumoto N Uchida K Kishino Y Suemoto CK Prevalence of behavioral and psychological symptoms in patients with cognitive decline before and during the COVID-19 pandemic. Front Psychiatry. (2022) 13:839683. 10.3389/fpsyt.2022.839683 35321225 PMC8934776

[47] Brown EE Kumar S Rajji TK Pollock BG Mulsant BH. Anticipating and mitigating the impact of the COVID-19 pandemic on Alzheimer's disease and related dementias. Am J Geriatr Psychiatry. (2020) 28(7):712–21. 10.1016/j.jagp.2020.04.010 32331845 PMC7165101

[48] Canevelli M Valletta M Toccaceli Blasi M Remoli G Sarti G Nuti F Facing dementia during the COVID-19 outbreak. J Am Geriatr Soc. (2020) 68(8):1673–6. 10.1111/jgs.16644 32516441 PMC7300919

[49] Morin CM Carrier J Bastien C Godbout R Canadian S Circadian N. Sleep and circadian rhythm in response to the COVID-19 pandemic. Can J Public Health. (2020) 111(5):654–7. 10.17269/s41997-020-00382-7 32700231 PMC7375451

[50] Steinman MA Perry L Perissinotto CM. Meeting the care needs of older adults isolated at home during the COVID-19 pandemic. JAMA Intern Med. (2020) 180(6):819–20. 10.1001/jamainternmed.2020.1661 32297903

[51] El Haj M Jardri R Laroi F Antoine P. Hallucinations, loneliness, and social isolation in Alzheimer's disease. Cogn Neuropsychiatry. (2016) 21(1):1–13. 10.1080/13546805.2015.1121139 26740416

[52] El Haj M Laroi F Gallouj K. Hallucinations and COVID-19: increased occurrence of hallucinations in patients with Alzheimer's disease during lockdown. Psychiatr Q. (2021) 92(4):1531–9. 10.1007/s11126-021-09927-6 34089149 PMC8178053

[53] Ferro Uriguen A Laso Lucas E Sannino Menicucci C Iturrioz Arrechea I Alaba Trueba J Echevarria Orella E Psychotropic drug prescription in nursing homes during the COVID-19 pandemic. Drugs Aging. (2022) 39(6):467–75. 10.1007/s40266-022-00948-9 35726042 PMC9208968

[54] Coe AB Montoya A Chang CH Park PS Bynum JPW Shireman TI Behavioral symptoms, depression symptoms, and medication use in Michigan nursing home residents with dementia during COVID-19. J Am Geriatr Soc. (2023) 71(2):414–22. 10.1111/jgs.18116 36349415 PMC9877723

[55] Yan D Temkin-Greener H Cai S. Did the COVID-19 pandemic affect the use of antipsychotics among nursing home residents with ADRD? Am J Geriatr Psychiatry. (2023) 31(2):124–40. 10.1016/j.jagp.2022.09.009 36272888 PMC9514966

[56] Maxwell CJ Dampf H Alkabbani W Cotton CA Gamble JM Hogan DB Psychotropic, anticonvulsant, and opioid use in assisted living residents before and during the COVID-19 pandemic. J Am Med Dir Assoc. (2024) 25(1):121–9. 10.1016/j.jamda.2023.09.009 37863111

[57] Centers for Medicare & Medicaid Services. National partnership to improve dementia care in nursing homes: antipsychotic medication use data report (2019). Available online at: https://www.cms.gov/Medicare/Provider-Enrollment-and-Certification/SurveyCertificationGenInfo/Downloads/Antipsychotic-Medication-Use-Data-Report.pdf (Accessed October 20, 2025). 26110197

[58] Reus VI Fochtmann LJ Eyler AE Hilty DM Horvitz-Lennon M Jibson MD The American Psychiatric Association practice guideline on the use of antipsychotics to treat agitation or psychosis in patients with dementia. Am J Psychiatry. (2016) 173(5):543–6. 10.1176/appi.ajp.2015.173501 27133416

[59] Korkatti-Puoskari N Tiihonen M Caballero-Mora MA Topinkova E Szczerbinska K Hartikainen S Therapeutic dilemma's: antipsychotics use for neuropsychiatric symptoms of dementia, delirium and insomnia and risk of falling in older adults, a clinical review. Eur Geriatr Med. (2023) 14(4):709–20. 10.1007/s41999-023-00837-3 37495836 PMC10447285

[60] Watt JA Porter J Tavilsup P Chowdhury M Hatch S Ismail Z Guideline recommendations on behavioral and psychological symptoms of dementia: a systematic review. J Am Med Dir Assoc. (2024) 25(5):837–46 e21. 10.1016/j.jamda.2024.03.007 38640961

[61] Hsu TW Stubbs B Liang CS Chen TY Yeh TC Pan CC Efficacy of serotonergic antidepressant treatment for the neuropsychiatric symptoms and agitation in dementia: a systematic review and meta-analysis. Ageing Res Rev. (2021) 69:101362. 10.1016/j.arr.2021.101362 34000464

[62] Dong M Liu C Luo H Su D Li G Xu F Efficacy and tolerability of antidepressants monotherapy for behavioral and psychological symptoms of dementia: a meta-analysis of randomized controlled trials. J Psychiatr Res. (2025) 181:417–24. 10.1016/j.jpsychires.2024.12.005 39662328

[63] Johnell K Jonasdottir Bergman G Fastbom J Danielsson B Borg N Salmi P. Psychotropic drugs and the risk of fall injuries, hospitalisations and mortality among older adults. Int J Geriatr Psychiatry. (2017) 32(4):414–20. 10.1002/gps.4483 27113813 PMC5347947

[64] Schnier C McCarthy A Morales DR Akbari A Sofat R Dale C Antipsychotic drug prescribing and mortality in people with dementia before and during the COVID-19 pandemic: a retrospective cohort study in Wales, UK. Lancet Healthy Longev. (2023) 4(8):e421–30. 10.1016/S2666-7568(23)00105-8 37543047

[65] Franchi B. Pharmacological management of behavioural and psychological symptoms of dementia. J Pharm Pract Res. (2016) 46(3):277–85. 10.1002/jppr.1260

[66] Rijksen DOC Zuidema SU de Haas EC. Use of benzodiazepines and Z-drugs in nursing home residents with dementia: prevalence and appropriateness. J Alzheimers Dis Rep. (2021) 5(1):871–9. 10.3233/ADR-210041 35088036 PMC8764627

[67] Tampi RR Tampi DJ. Efficacy and tolerability of benzodiazepines for the treatment of behavioral and psychological symptoms of dementia: a systematic review of randomized controlled trials. Am J Alzheimers Dis Other Demen. (2014) 29(7):565–74. 10.1177/1533317514524813 25551131 PMC10852883

[68] De Crescenzo F D'Alo GL Ostinelli EG Ciabattini M Di Franco V Watanabe N Comparative effects of pharmacological interventions for the acute and long-term management of insomnia disorder in adults: a systematic review and network meta-analysis. Lancet. (2022) 400(10347):170–84. 10.1016/S0140-6736(22)00878-9 35843245

[69] Shen K Loomer L Abrams H Grabowski DC Gandhi A. Estimates of COVID-19 cases and deaths among nursing home residents not reported in federal data. JAMA Network Open. (2021) 4(9):e2122885. 10.1001/jamanetworkopen.2021.22885 34499136 PMC8430452

[70] White EM. Underreporting of early nursing home COVID-19 cases and deaths in federal data. JAMA Network Open. (2021) 4(9):e2123696. 10.1001/jamanetworkopen.2021.23696 34499139 PMC9002925

