# Supplementary material for: Behavioral symptoms of dementia and psychotropic use during the COVID-19 pandemic
Source: Front Epidemiol. 2026 Apr 13;6:1746012. doi: 10.3389/fepid.2026.1746012 (PMC13111391; doi:10.3389/fepid.2026.1746012)
Supplement: Supplementary file 1 [file Table1.docx]

**SUPPLEMENTARY MATERIAL**

Behavioral Symptoms of Dementia and Psychotropic Use During the COVID-19 Pandemic. *Frontiers in Epidemiology*. XXXX;X():eXXXXX.

This supplemental material has been provided by the authors to give readers additional

information about their work.

**eTable S1:** List of medical comorbidities included in analyses

**eTable S1:** List of medical comorbidities included in analyses

| Cancer | Arthritis |
| --- | --- |
| Anemia | Osteoporosis |
| Cardiac dysrhythmia | Hip fracture |
| Coronary artery disease (CAD) | Other fracture |
| Deep vein thrombosis (DVT) | Thyroid disorder |
| Heart failure | Aphasia |
| Hypertension | Cerebral palsy |
| Hypotension | Stroke |
| Peripheral vascular disease (PVD) | Respiratory failure |
| Cirrhosis | Hemiplegia |
| Gastroesophageal reflux disease (GERD) | Paraplegia |
| Ulcerative colitis | Quadriplegia |
| Benign prostatic hyperplasia (BPH) | Multiple sclerosis (MS) |
| End-stage renal disease (ESRD) | Huntington’s disease |
| Neurogenic bladder | Parkinson’s disease |
| Obstructive uropathy | Tourette’s syndrome |
| Multidrug-resistant organism (MDRO) | Seizure disorder |
| Pneumonia | Brain injury |
| Septicemia | Malnutrition |
| Tuberculosis (TB) | Anxiety disorder |
| Urinary tract infection (UTI) | Asthma |
| Viral hepatitis | Manic depression (bipolar disorder) |
| Wound infection | Other psychiatric condition |
| Diabetes mellitus (DM) | Schizophrenia |
| Hyponatremia | Post-traumatic stress disorder (PTSD) |
| Hyperkalemia | Cataracts |
| Hyperlipidemia |  |
